# Ecological Building Material Obtained Through the Moderate Thermal Consolidation of Ceramic Slurry Collected from Industrial Waste Waters

**DOI:** 10.3390/ma18081715

**Published:** 2025-04-09

**Authors:** Simona Elena Avram, Bianca Violeta Birle, Cosmin Cosma, Lucian Barbu Tudoran, Marioara Moldovan, Stanca Cuc, Gheorghe Borodi, Ioan Petean

**Affiliations:** 1 Faculty of Materials and Environmental Engineering, Technical University of Cluj-Napoca, 103-105 Muncii Bd., 400641 Cluj-Napoca, Romania; simona.avram@imadd.utcluj.ro (S.E.A.); birle.ma.bianca@student.utcluj.ro (B.V.B.); 2 Department of Manufacturing Engineering, Machine Building, Technical University of Cluj-Napoca, 400641 Cluj-Napoca, Romania; cosmin.cosma@tcm.utcluj.ro; 3 Faculty of Biology and Geology, Babes-Bolyai University, 44 Gheorghe Bilaşcu Street, 400015 Cluj-Napoca, Romania; lucian.barbu@ubbcluj.ro; 4 National Institute for Research and Development of Isotopic and Molecular Technologies, 65-103 Donath Street, 400293 Cluj-Napoca, Romania; borodi@itim-cj.ro; 5 Department of Polymer Composites, Raluca Ripan Institute for Research in Chemistry, Babeș-Bolyai University, 30 Fantanele Street, 400294 Cluj-Napoca, Romania; marioara.moldovan@ubbcluj.ro (M.M.); stanca.boboia@ubbcluj.ro (S.C.); 6 Faculty of Chemistry and Chemical Engineering, Babes-Bolyai University, 11 Arany Janos Street, 400028 Cluj-Napoca, Romania

**Keywords:** ceramic slurry, dehydrated kaolinite, ecological bricks

## Abstract

The slurry collected from the waste water resulting from ceramic tile processing contains significant amounts of quartz, kaolinite, and mullite, along with traces of iron hydroxides as observed using XRD analysis coupled with mineralogical optical microscopy (MOM). Such an admixture would be ideal for the development of ecologic building materials. Microstructural conditioning enhances the binding properties of kaolinite. Therefore, the influence of the vibration compaction of the moistened slurry at 30% humidity on the compressive strength was assessed. The compressive strength of the unvibrated sample is about 0.8 MPa with failure promoted by the microstructural unevenness. Several vibration amplitudes were tested from 20 to 40 mm. The optimal vibration mode was obtained at an amplitude of 25 mm for 10 min, ensuring a compressive strength of 2.37 MPa with a smooth and uniform failure surface involved within the binding layer as observed using SEM microscopy. The samples prepared under optimal conditions were thermally consolidated at 700, 800, and 900 °C below the mullitization temperature to ensure a low carbon footprint. XRD results reveal kaolinite dehydration in all fired samples, inducing its densification, which increases with increasing heating temperature. SEM coupled with EDS elemental investigations reveal that the dehydrated kaolinite better embeds quartz and mullite particles, ensuring a compact microstructure. The binding strength increases with the firing temperature. The mullite particles within the samples fired at 900 °C induce the partial mullitization of the dehydrated kaolinite matrix, increasing their homogeneity. The compression strength of the fired samples is temperature dependent: 4.44 MPa at 700 °C; 5.88 MPa at 800 °C, and 16.87 MPa at 900 °C. SEM fractography shows that failure occurs due to the dehydrated kaolinite matrix cracks and the quartz particles. The failure is rather plastic at low temperatures and becomes brittle at 900 °C. Reducing the firing temperature and treatment time reduces the carbon footprint of the consolidated ceramic parts. Samples fired at 700 °C exhibit a compressive strength comparable to low quality bricks, those fired at 800 °C exhibit a strength comparable to regular bricks, and those fired at 900 °C exhibit a superior strength comparable to high-quality bricks.

## 1. Introduction

Sustainable materials should conserve the natural resources of Earth, providing advanced ecological solutions for the development of the modern lifestyle. Such a lifestyle commonly implies a very polluting industry generating large number of wastes [1,2] and consuming huge amounts of energy, increasing the carbon footprint [3,4,5]. Therefore, the development of sustainable materials should recover a significant number of wastes, reducing the amount sent to landfills and dumps [6,7], and should be processed at a minimum acceptable temperature, reducing their carbon footprint [8,9].

The ceramic tile industry is a high energy consumer, generating significant amount of solid wastes, which are usually re-circulated in the same technological process due to their compositional compatibility [10,11]. Unfortunately, the ceramic slurry collected from their used waters cannot be re-circulated due to its contamination with iron hydroxides, which causes glaze stains [12,13]. Their dumping in landfills requires special care, including periodical wetting to prevent particulate matter emissions into atmosphere [14]. An alternative recycling mode for this potentially hazardous ceramic slurry must be found. Our previous studies reveal that the proper conditioning of the kaolinite matrix by exerting external pressure [15] and by ensuring a proper moistening around 30% humidity [16] ensures increased compressive strength about of 1 to 1.5 MPa and a flexural strength of 0.5 to 1.2 MPa. These achievements are useful for the further development of sustainable materials. The reinforcing of Stipa Pennata with natural fibers ensures a significant increase in the compressive strength in a range of 3–5 MPa depending on the orientation of the fibers within the reinforcing layer and their position in the sample [17]. It allows the development of ecologically reinforced panels to cover walls, but their mechanical properties are far below the requirement for wall construction. Another path needs to be followed to achieve enough strength and cohesion to develop structural units for building, such as bricks.

Classical brick production requires firing temperatures between 700 and 1100 °C, depending on the clay feedstock and desired quality (e.g., compressive strength). Therefore, low-quality bricks have a compressive strength of 3.5 MPa, regular quality bricks are situated around 6 MPa, and that of first-class bricks is about 10 MPa [18]. It is a matter of the mullitization process occurring at high temperatures. The clay mixture commonly used for the brick’s fabrication contains kaolinite and muscovite. Kaolinite transforms into mullite at temperatures ranging from 1000 to 1300 °C, depending on the mineral purity, its interaction with quartz, and its transformation into cristobalite [19]. Thus, according to the literature, the mullitization temperature of clay is about 1100 °C [20,21]. On the other hand, muscovite starts to transform into mullite at 900 °C by losing the hydroxyl groups within the crystalline structure and is finalized at a temperature of about 1100 °C [22,23]. Rehman et al. reveals that oxide waste products adding to the brick’s mixture reduce the sintering temperature by 50–100 °C given that their fluxing within the granular material enhances their consolidation [24]. Other research uses glass wastes to reduce the consolidation temperature of the developed bricks [25].

Broad research indicates that waste incorporation into the structure of bricks might be effectuated by firing, cementing, and geopolymerization. The optimal method depends on waste characteristics: clay like composition is suitable for firing, granular matter such as fly ash is suitable for geopolymerization, and recycled aggregates such as reconverted mortars are suitable for cementation [26,27,28]. Our previous studies [15,16,17] reveal that the slurry collected from used water resulting from the fabrication of ceramic tiles contains mainly quartz, kaolinite, and mullite, as well as traces of iron hydroxide. The novelty of the current investigation involves the preparation of a sustainable material produced using thermal consolidation with a low carbon footprint that promotes kaolinite matrix dehydroxylation. The presence of mullite particles could act as sintering promoters under certain conditions, allowing the achievement of proper thermal consolidation at temperatures less than 1100 °C, which are required for standard mullitization [20,21]. The actual brick configuration is relatively complicated because of the channel’s geometry. The kaolinite matrix conditioning obtained by applying the compaction load during molding cannot be applied in such conditions. Therefore, a pasty consistence of the molding mixture is required. This can be obtained with 30% humidity, allowing the inter-motion of quartz and mullite particles within kaolinite mixture. The exposure of molded samples to vibration might ensure proper microstructural compaction, allowing further exposure to thermal consolidation. Thus, the first step of the current research is establishing the optimal vibration method for the compaction of the molded samples, and the second step is the thermal consolidation of the vibrated samples under optimal conditions. Several temperatures in the range of 700–900 °C are required to be investigated to observe the crystalline phases, microstructural changes, and their influence on the mechanical properties.

The minerals changes induced by firing are followed using X ray diffraction (XRD) correlated with mineralogical optical microscopy (MOM), while the microstructural aspects are investigated using scanning electron microscopy coupled with energy dispersive spectroscopy, revealing the elemental composition of the microstructural features. The compressive strength is monitored for all involved samples and correlated with fractography images obtained using SEM. The complex physicochemical investigation of the samples associated with compressive strength measurements allows observations of the specific characteristics of the obtained material at each temperature, proving its sustainability. Considering the heat treatment regime for each sample, the carbon footprint can be established.

## 2. Materials and Methods

### 2.1. Samples Preparation

The used slurry was recycled from the waste water of a ceramic tile production facility in Cluj County, Romania (the company name is kept anonymous for economic reasons). It consists of a dense paste that was collected in 10 L plastic receptacles at ambient temperature and transported to the laboratory where it was further processed.

Our previous studies reveal that the collected slurry has microstructural unevenness that affects the cohesion and mechanical properties of the raw compacts [15,17], requiring proper conditioning. As a consequence, the slurry humidity was adjusted to 30% using technological water (used water resulting from the technological process used to produce wall and floor tiles with alkaline characteristics of pH = 10) and milled at 6000 rpm in a blade mill for 5 min.

The conditioned slurry was molded into cylindrical vials with a diameter of 12 mm (the vials walls have a small inclination of 0.5%/cm for easy de-molding) at a height of 12 mm. The molded samples were vibrated for 10 min on a vibratory table at different amplitudes: 0, 20, 25, 30, 35, and 40 mm.

**Figure 1:**
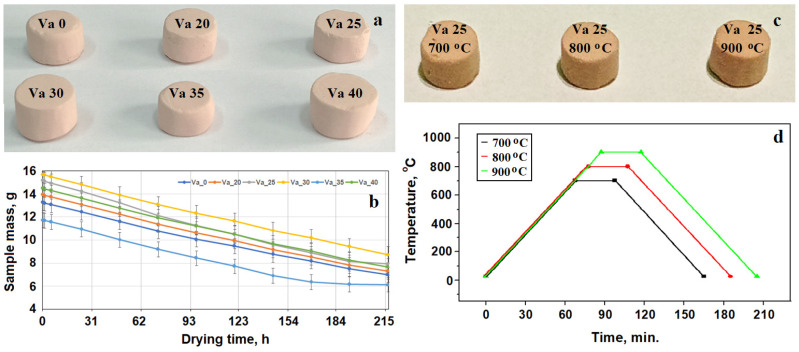
Sample photographs and thermal treatment diagrams: (**a**) vibrated samples, (**b**) drying diagram of vibrated samples, (**c**) photograph of fired samples, and (**d**) firing diagram.

The vibrated samples were naturally dried in the mold for 4 days and extracted afterwards. Their shape was adjusted to a diameter of 10 mm and height of 11 mm, with the general aspect depicted in Figure 1a. Their mass was measured periodically during the 4 days of natural drying, as shown in Figure 1b. The adjusted samples were stored for three months under ambient conditions prior to mechanical properties testing.

The experimental results show that the best mechanical properties were obtained by vibrating at an amplitude of 25 mm. A lot of nine identical specimens were prepared under the best condition and divided into three samples that were fired at 700, 800, and 900 °C. Their general aspect is depicted in Figure 1c, and the firing diagram is presented in Figure 1d. The main characteristics of the sample’s preparation are centralized in Table 1.

Sample firing was effectuated in a resistive electric furnace N31/H (Nabertherm, Lilienthal, Germany) having a temperature uniformity of ±10 °C. They were introduced at room temperature under ambient atmosphere, heated in the furnace at a rate of 10 °C/min, maintained for 30 min at the desired temperature (Table 1), and cooled in the furnace at a rate of 10 °C/min. The fired samples were stored in the desiccators until they were subjected to compression testing.

### 2.2. Characterization Methods

The evolution of the mineral phase was investigated with X-ray diffraction (XRD) which was performed with a Bragg—Brentano diffractometer Bruker D8 Advance (Bruker Company, Karlsruhe, Germany) using Cu Kα radiation λ = 1.54056 A at a speed of 1°/min in the 2θ range of 10–80 degrees. The peak assignment and phase identification were effectuated with Match 1.0 software (Crystal Impact Company, Bonn, Germany) equipped with the PDF 2.0 database. The following PDF (Powder Diffraction Files) were used for the peak’s assignment: kaolinite 02-0125; lepidocrocite 76-2301; hematite 89-7047; mullite 06-0259; and quartz 89-8943. XRD data were correlated with the mineralogical optical microscopy (MOM) effectuated with a Laboval 2 microscope (Carl Zeiss, Oberkochen, Germany) operated in cross polarized light mode. The image was acquired digitally using a Sony 14 MPx (Sony Group Corporation, Minato, Japan).

Microstructural aspects were revealed using scanning electron microscopy (SEM) coupled with energy dispersive spectroscopy (EDS) effectuated with a Hitachi SU8230 (Hitachi Company, Tokyo, Japan) equipped with an X-Max 1160 EDX element detector (Oxford Instruments, Oxford, UK). The investigation was performed in high vacuum mode at an acceleration voltage of 30 kV. The sample’s surface was sputtered with a thin film of gold, ensuring optimal electrical conduction and allowing viewing of the high-resolution aspect of the microstructure. The gold component was subtracted during the elemental analysis.

Compression strength was measured with a Lloyd LR5k Plus testing machine (Ametek Lloyd Instruments, Meerbusch, Germany) with a progressive load of 0.5 N applied with a rate of 0.2 mm/min. Three specimens were tested for each sample, and the mean values of the compressive strength and compressive Young modulus was calculated. Their statistical analysis was performed with Microcal Origin Lab version 2018b software (Microcal Company, Northampton, MA, USA) using ANOVA tests followed by Tukey post hoc test at a significance level *p* = 0.05.

The sample’s fracture surface was investigated with SEM using an Inspect S (FEI Company, Hillsboro, OR, USA) in low vacuum mode at an acceleration voltage of 25 kV. The fracture surface porosity was assessed using the SEM images of the fracture surface with Image J software version 1.53t (National Institute of Health, Bethesda, Rockville, MD, USA), and the pore distribution was assessed according to their diameter and total porosity.

## 3. Results

### 3.1. Evolution of Mineral Phases 

**Figure 2:**
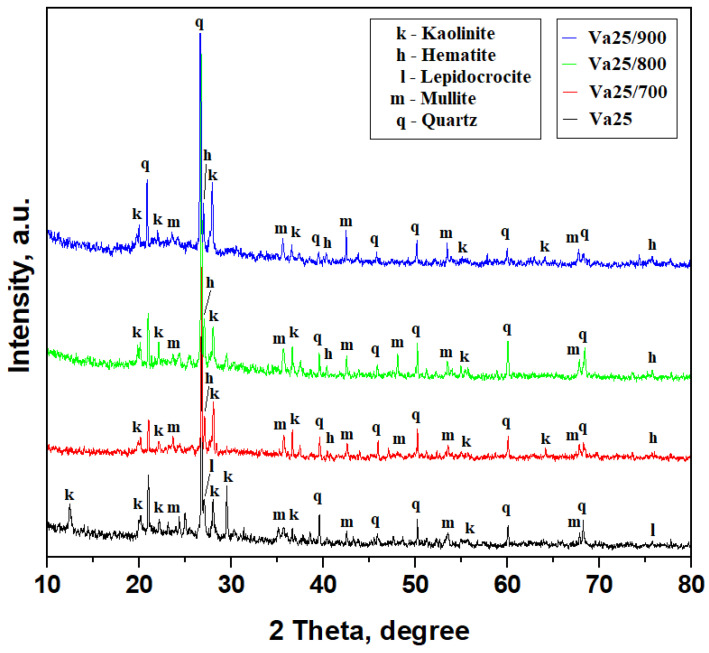
XRD patterns of the samples investigated.

The Va25 sample has a composite texture dominated by the quartz particles acting as filler accompanied by moderate amounts of mullite and kaolinite particles acting as a matrix embedding small amounts of lepidocrocite. Quartz’s well-developed and narrow diffraction peaks support this fact, while kaolinite peaks are rather broadened and less intense because of their small particles, as shown in Figure 2. Mullite peaks are narrow and less intense because of the relatively low amount in the sample. Lepidocrocite peaks are weak due to their low amount and difficult to observe among the other stronger peaks; however, the crystal plane (126) is well evidenced by the peak at 27.13°. Each mineral amount was established using the reference intensity ratio (RIR) method previously described in [16]. It was applied on the XRD patterns, and the obtained values are presented in Table 2. The results show that Va25 has a typical mineral composition for very well-conditioned ceramic slurry as observed in our previous studies [15,17].

Heat treatment induces major changes within the kaolinite matrix because it affects its internal structure. Thus, the kaolinite peaks evidenced at 12.33° and 29.59° are affected by the adsorbed water within the (001) and (−112) planes, which is not completely released during the drying procedure. These crystallographic planes are also related to the OH bonds within the kaolinite structure. We observe their disappearance after heat treatment at 700 °C, as shown in Figure 2, along with a progressive increase in the relative intensity of the kaolinite peaks at 27.90° corresponding to the (111) plane and 36.35° corresponding to the (−112) plane as the heating temperature increases for Va25/700, Va25/800, and Va25/900. These features correspond to the kaolinite dehydration inducing densification of the kaolinite matrix over the filler particles, and this is in good agreement with the literature data [29,30]. One form of dehydroxylated kaolinite is known as metakaolin, and it has a rather amorphous structure that is very useful for the pozzolanic effect. However, this is not the case here.

The relevant characteristic diffraction peaks in Figure 2 provide evidence of the preexistent mullite particles. Some of them partly overlap the quartz and kaolinite peaks due to the presence of SiO_2_ tetrahedra in their structure, but several representative non-overlapping mullite peaks are found at 23.21° for (200), 35.56° for (060), 42.52° for (361), and 42.43° for the crystallographic plane (361). The mullite peak intensities for the planes (060) and (361) increase with the firing temperature, indicating an appreciation of its amount in the thermal consolidated samples, especially Va25/900.

Iron hydroxide (as the most degraded form of iron oxides) is found as lepidocrocite in the Va25 sample in good agreement with our previous observations [16,17]. The literature data reveal that lepidocrocite heating over 250 °C transforms it into Fe_2_O_3_ as hematite [31,32]. The fact is very well observed by the transformation of lepidocrocite crystallographic plane (126) into hematite (111) by a slightly displacement from 27.13° to 26.94°, as shown in Figure 2.

The RIR method was applied for all XRD patterns in Figure 2, and the mineral phase amount was determined for each temperature, as shown in Table 2. It is observed that the quartz amount is constant regardless of temperature. Lepidocrocite and hematite amounts are also constant within the samples. The transformation of kaolinite into dehydroxylated kaolinite along with the temperature increase implies a significant decrease in its amount proportionally with increase in mullite.

**Figure 3:**
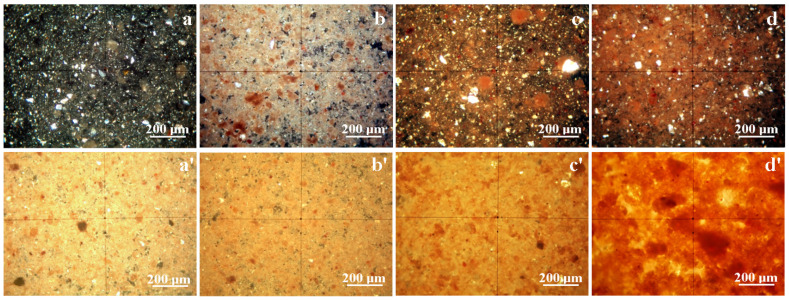
MOM images of the thin layer of the investigated samples: (**a**) Va25, (**b**) Va25/700, (**c**) Va25/800, and (**d**) Va25/900. The thick layer observed using MOM for each sample: (**a′**) Va25, (**b′**) Va25/700, (**c′**) Va25/800, and (**d′**) Va25/900.

Thin layer assessment of Va25 reveals quartz particles having boulder-like shapes that are green and gray in color accompanied by preexistent mullite particles with rounded boulder shapes that are orange and brown in color embedded in the kaolinite matrix composed of fine tabular and lamellar kaolinite particles that are white and pale blue in color, as shown in Figure 3a. Some small red-brown particles are observed, representing iron hydroxide crystallized as lepidocrocite. The MOM image in Figure 3a allows the establishment of each mineral particle’s size range in Va25, as shown in Table 2. The thick layer, as shown in Figure 3a′, is more representative of the green slurry compact within Va25, revealing a dense distribution of the kaolinite matrix with small particles randomly oriented, giving a uniform white-yellow aspect surrounding the quartz and mullite filler particles.

Heating at 700 °C causes significant transformation within Va25/700 samples. Kaolinite dehydroxylation densifies the samples matrix with a slight appreciation of the particle size due to their relative coalescence, and their specific color turns white and yellow, as shown in Figure 3b. Mullite particles are more evident in the thin layer section, having slightly increased in size. It is worth noting that quartz particles also have a yellow color because of thermal treatment, but it did not affect its crystal structure. The intense red-brown spots appear more concentrated, indicating the transformation of lepidocrocite into hematite grains. VA25/700’s thick layer, as shown in Figure 3b′, has a similar appearance to Va25, but the matrix has a more intense yellow aspect as consequence of kaolinite dehydration.

Major changes occur in Va25/800 as consequence of high temperature action. Quartz particles maintain their size and shape as well as a specific yellow tarnish, but the size of the mullite particles significantly increases given the dense contact with the dehydrated kaolinite matrix. This causes it to turn orange, indicating advanced loss of the OH bonds within the crystal structure, as shown in Figure 3c. The dehydrated kaolinite densification causes the finest particles to cluster, inducing a relative increase in their size. This fact influences the thick layer of Va25/800. It has pronounced mullite particles tightly embedded into the dehydroxylated kaolinite matrix, which has an intense yellow appearance, as shown in Figure 3c′.

Heat treatment of the Va25/900 sample induces severe modifications of the mineral phases, as shown in Figure 3d. Mullite particles significantly increase in size because of the promotion of diffusion necks, indicating the start of sintering. The dehydroxylated kaolinite matrix achieves the highest microstructural density and tightly surrounds the mullite particles in a very cohesive manner. The Quartz particles reinforce this consolidated ceramic structure, ensuring the balance and material uniformity of the filler particles. The red spots are more visible because of the stronger segregation of the iron oxide, confirming the literature information regarding the formation of glaze stains through hematite clustering [12,13]. The thick layer of Va25/900 better evidences the sintering connection of the mullite particles as a spatial network embedded into the dehydroxylated kaolinite matrix that tightly surrounds the quartz particles.

The used water slurry obtained from the fabrication of ceramic tiles contains moderate to low amounts of glass particles derived from the preparation of raw materials. Their purpose is to fuse at relatively low temperatures, ensuring microstructural cohesion of the finest particles prior to kaolinite dehydration. These glass particles are amorphous and cannot be detected in the XRD patterns. However, MOM observation identify them as dark particles in Figure 3a with a broken aspect due to their advanced milling under raw materials preparation. Therefore, their size ranges from about 5 to 30 μm. They completely disappear in the samples heated to high temperatures because they fuse and mix with fine kaolinite particles and are subsequently embedded in the matrix body. Their melted traces might be found by tracing Ca and Mg atoms within glass chemical composition where these elements act as melting mediators [33,34]. However, this requires detailed elemental mapping.

### 3.2. Microstructural Aspects and Elemental Composition

**Figure 4:**
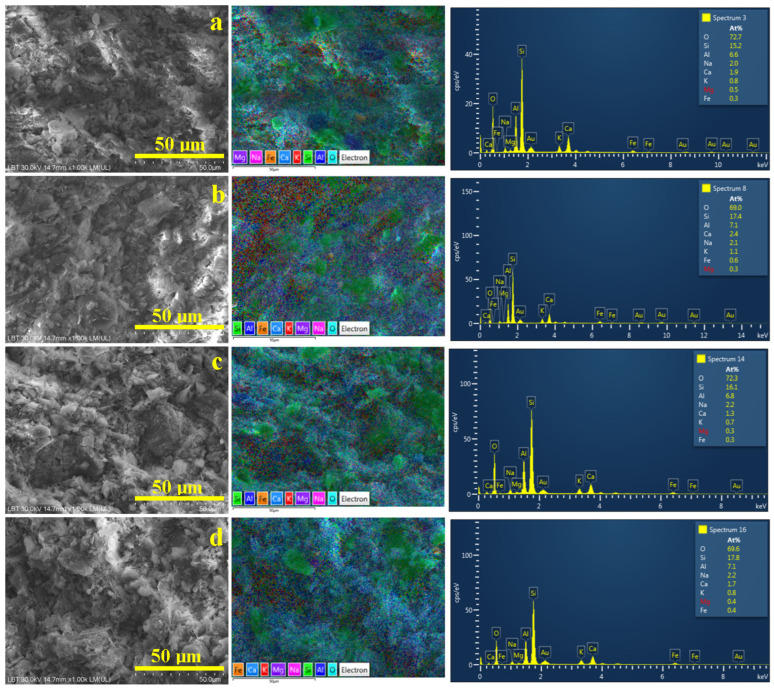
SEM images of the samples investigated: (**a**) Va25, (**b**) Va25/700, (**c**) Va25/800, and (**d**) Va25/900. The elemental map and EDS spectrum are presented on the right side of each image.

The optimal view of the sample microstructure is provided in the scanning electron microscopy (SEM) images coupled with elemental mapping, as shown in Figure 4. The elemental map detects the atom species on the sample’s surface and displays them with distinct colors that are overlaid on the microstructural details evidenced in the SEM image. In our case, silicon is labeled in green, aluminum is labeled in dark blue, oxygen is labeled turquoise, potassium in red, and iron in orange. The trace elements related to glass particles are labeled as follows: Ca—light blue, Mg—deep violet, and Na—pink. Therefore, quartz particles appear as green boulders while mullite particles look like dark blue rounded boulders. Kaolinite and dehydroxylated kaolinite matrix appear blue with some red spots surrounding the filler particles. Iron is finely dispersed within kaolinite matrix in the Va25 sample, as shown in Figure 4a. However, iron tends to form small orange clusters in the VA25/700 sample, as shown in Figure 4b, and become more concentrated in the samples fired at 800 and 900 °C, as shown in Figure 4c,d.

The elemental composition of the samples is presented in Table 3. Oxygen is the dominant element because it is found in all the mineral components of the samples.

Silicon is the second most common element because most of the minerals within the ceramic slurry belong to silicate group. Aluminum is the most important element in kaolinite and mullite because it ensures the connection between the Si-O tetrahedral sheets forming the octahedral layer [35]. However, Al’s trivalent nature allows exchanging K^+^ ions with its geological environment, explaining the small amounts of K detected in all samples. The amount of iron detected confirms the XRD observation regarding the presence of lepidocrocite and hematite in the Va25 sample and heat-treated samples. The data in Table 3 show a constant composition of the main elements within all samples, indicating that temperature changes are only physically inducing kaolinite restructuration in dehydroxylated kaolinite and mullite without chemical loss of the main elements.

We observe that Ca traces are significantly reduced while Na traces are slightly increased within the sample’s microstructure, thus constantly maintaining the Mg level t. It indicates the melting of glass particles through the kaolinite matrix. Such behavior confirms the melting of glass particles and the distribution of Ca atoms in the dehydroxylated kaolinite matrix. The Ca atom distribution mechanism is sustained by the concentrated pale blue regions within the elemental map in Figure 4a, indicating the presence of glass particles and its uniform distribution within the dehydroxylated kaolinite matrix in the elemental maps in Figure 4b–d.

**Figure 5:**
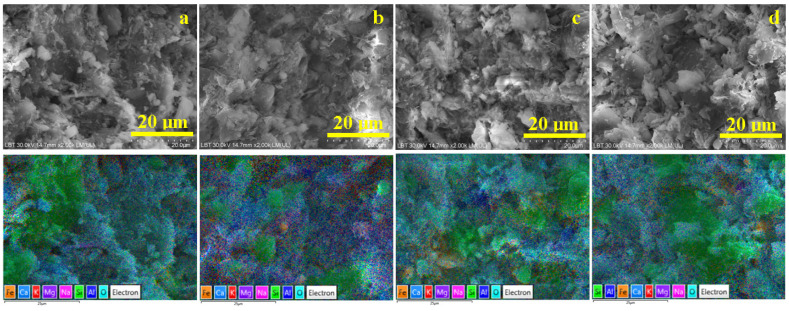
SEM microstructural details of the embedding layer within the samples investigated: (**a**) Va25, (**b**) Va25/700, (**c**) Va25/800, and (**d**) Va25/900.

The microstructural detail in Figure 5a reveals the optimal compaction of the sample vibrated at 25 mm for 10 min. Quartz and mullite filler particles are well dispersed and surrounded by lamellar and tabular kaolinite particles and some fine glass fractions. There are mostly finest kaolinite particles that are 2–5 μm in size and fewer bigger sheets of about 15 μm with a thickness of about 5 μm. These matrix particles are well adsorbed onto the filler particles, providing good cohesion, and this should ensure fair compression strength.

The thermal consolidation effectuated by heating at 700 °C first induces the melting of glass particles, which enhances the adhesion of kaolinite particles on quartz and mullite filler particles within Va25/700. The kaolinite dehydroxylation turns the matrix in a compact structure, indicating the clustering of fine particles welding the lamellar and tabular plates of 15 μm into a compact structure, as shown in Figure 5b. Similar aspects occur in Va25/800. However, the dehydroxylated kaolinite-densified platelets are about 20 μm, and thickness varies from 5 to 8 μm, as shown in Figure 5c.

The sample heated at 900 °C has a more densified dehydroxylated kaolinite matrix with bigger tabular platelets of about 20 μm strongly attached to the quartz particles and deeply fused with the pre-existent mullite filler particles. It indicates partial transformation of the dehydroxylated kaolinite into mullite at the contact interfaces, sintering the adjacent particles into a compact microstructure, as shown in Figure 5d. The uniform aspect of the embedding matrix within the elemental map supports this fact. Such a microstructure should ensure superior compressive strength, which must be further investigated.

### 3.3. Mechanical Properties

**Figure 6:**
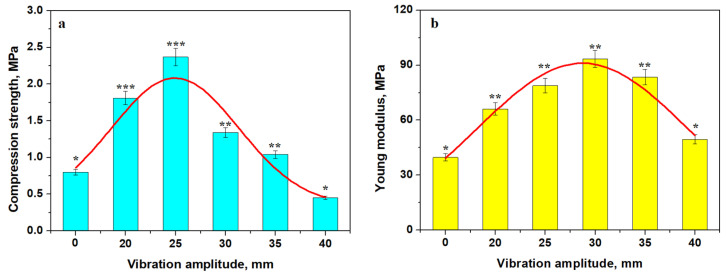
The variation in mechanical properties with the vibration’s amplitude: (**a**) compression strength and (**b**) Young’s modulus. The relevant statistical groups are marked with: (*) first group, (**) second group and (***) third group. The red lines are the variation trend.

The green compact cohesion is ensured by a proper conditioning of the kaolinite matrix, which was exerted by the external pressure applied just after molding [16,17]. The complicated geometry of the molded bricks might impede the exertion of the external pressure necessary for proper slurry compaction. Therefore, vibrating the freshly molded sample could facilitate the proper microstructural compactness. The vibration amplitude was set on the vibratory devices and range from 20 to 40 mm in 5 mm increments. The optimal value must be found based on experimental evidence. The dependence of the resulting compressive strength on the vibration amplitude is presented in Figure 6.

The compression strength variation has a Gaussian variation with a maximum at an amplitude of 25 mm. Thus, the Va25 sample has the best strength, while Va0 and Va40 have the worst compressive strength, as shown in Figure 6a. The Young’s modulus has a similar Gaussian variation, as shown in Figure 6b, with a maximum value for Va30 followed by Va35 and Va25, indicating good elastic behavior of the compacted structure. Va0 and Va40 have the worst behavior.

The statistical analysis was effectuated with ANOVA and Tukey post hoc tests, as shown in Figure 6a. It reveals three distinct statistical groups for the compressive strength values having *p* values below the significance level of 0.05. The first group contains Va0 and Va40 characterized by low compressive strength (*p* > 0.05). The second group contains Va30 and Va35 characterized by a slightly increased compressive strength, and their mean values generate a *p* > 0.05. Finally, the third statistically relevant group contains Va20 and Va25 having strongly increased compressive strength (*p* > 0.05).

The Young modulus variation in Figure 6b also presents two relevant statistical groups. The first group is characterized by a low elasticity modulus value for Va0 and Va40 (*p* > 0.5). The second relevant group has increased elastic modules containing Va20, Va25, Va30, and Va35 (*p* > 0.05). Statistical analysis reveals relevant differences between these two groups (*p* < 0.05).

**Figure 7:**
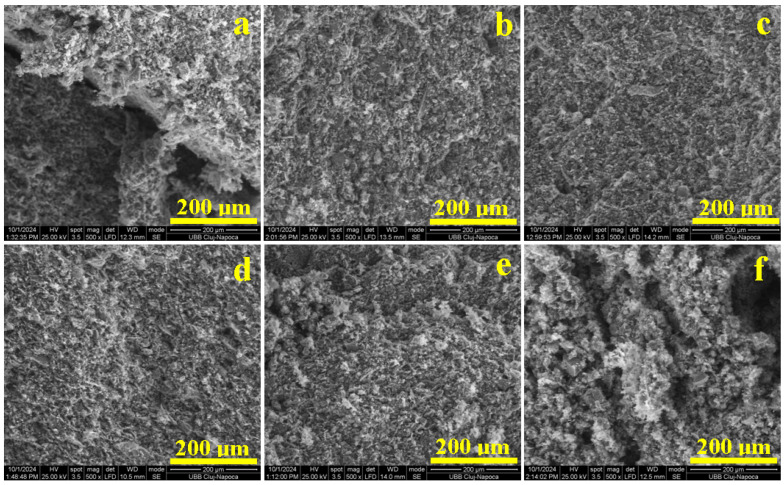
SEM images of the fracture surface of the vibrated samples: (**a**) Va0, (**b**) Va20, (**c**) Va25, (**d**) Va30, (**e**) Va35, and (**f**) Va40.

The behavior of the green compact is explained using fracture surface assessment with SEM, as shown in Figure 7. The Va0 sample presents an irregular fracture (Figure 7a) induced by microstructural pores and unevenness that weakens the material’s ability to dissipate the compressive effort. Therefore, the pores walls break under the compression load, causing sample failure. Similar failure is observed for the Va40 sample, evidencing pores ranging from 20 to 200 μm, as show in Figure 7f. A vibration amplitude of 40 mm facilitates the microstructural disorganization of the molded sample. The insufficient microstructural cohesion occurring within the Va0 and Va40 samples weakens the multi-planes of the kaolinite matrix, and this is in good agreement with Zhang’s observations [36]. The kaolinite matrix instability induces the relative displacement of aggregate particles within the failure surface, decreasing the compressive strength of the sample. This behavior was also reported by Wu [37].

Vibrations with an amplitude of 20 mm induce proper organization of the kaolinite particles, specifically quartz and mullite, ensuring a proper microstructural cohesion that properly resists under compressive effort, as demonstrated in the Va20 sample. The fracture surface has a uniform aspect without pores, as shown in Figure 7b, and only few dimples ranging from 5 to 10 μm occur where the kaolinite matrix was excoriated during the sample’s failure. The excoriations occur due to relative cleavage of the octahedral sheets containing Al atoms within the kaolinite foils. The filler particle’s binding is not affected by the proper conditioning of the matrix. A better aspect is observed for Va25 in Figure 7c, where the excoriating dimples are the finest (1–5 μm) and the fracture surface is smoother. This advanced microstructural compactness explains the best compression strength obtained by the uniform dissipation of the effort through the kaolinite adhesion layer.

**Figure 8:**
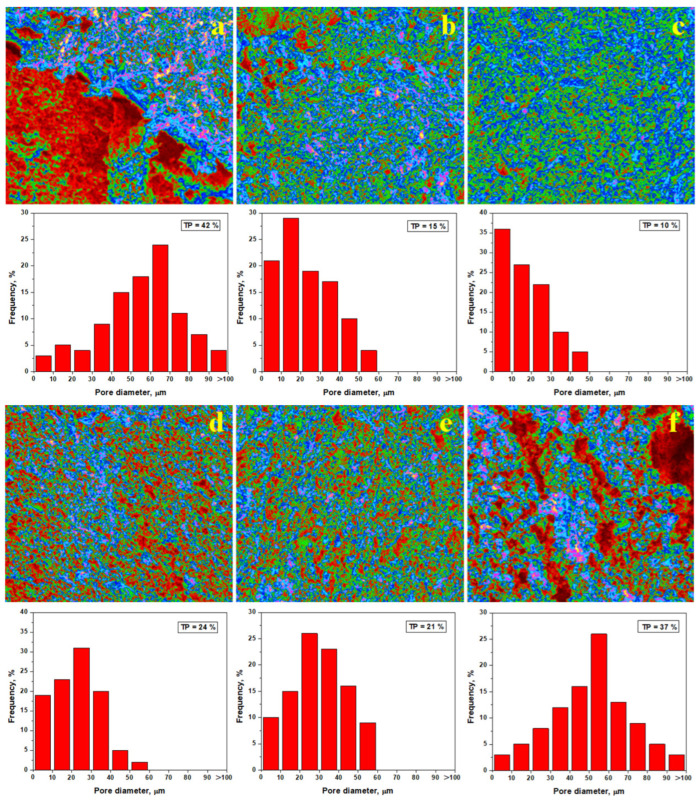
Pore distribution within the fracture surface of the vibrated samples: (**a**) Va0, (**b**) Va20, (**c**) Va25, (**d**) Va30, (**e**) Va35, and (**f**) Va40. TP—total porosity.

By increasing the amplitude vibration to 30 mm, the microstructural constituents gain too much freedom, which generates small micro-pores ranging from 5 to 10 μm uniformly distributed in the surface fracture, as shown in Figure 7d. The compression effort induces lateral stress within these small micro-pores, facilitating easy deformation of the kaolinite particles, delaminating quartz and mullite filler, and promoting failure. The internal pores increase in size in the Va35 sample. Most of them are clearly visible on the upper left side of Figure 7e and are about 10–30 μm. The compressive effort tends to deform these pores, altering the binding efficiency of the kaolinite matrix and causing a slightly irregular fracture that lowers the compressive strength. A quantitative investigation of the pores within the failure surfaces observed in Figure 7 was effectuated, resulting in the findings presented in Figure 8.

It is very difficult to assess the porosity of compact bodies because of the sealed pores inside of the sample’s bulk that cannot be detected using a standard mercury porosimeter. It would be necessary to have a helium pycnometer or use a micro CT technique [38,39], and these are not currently available for our research. Hu employed Image J-assisted quantification of the micro CT images of the porous structure [39]. On the other hand, Dong and collaborators developed an advanced pore quantification method based upon SEM imaging and EDS mapping, revealing smaller, coarse pores within the investigated structures [40].

Hence, the elemental mapping of the slurry samples was effectuated and correlated with the mineralogical aspects as revealed by XRD and mineralogical optical microscopy. Thus, information on the microstructural nature of the tested samples is obtained. The SEM images of the failure surface in Figure 7 show all pores. However, some of them are less visible with the naked eye but are visible using Image J software. Here, the pores are displayed in red color, the kaolinite matrix appears in green, and aggregate particles (quartz and pre-existent mullite particles) appear in blue, as shown in Figure 8. The weaker planes reported by [36] appear as pink faults. The pore diameter distribution histograms are presented for each analyzed image, and the total porosity is determined.

The first statistical group (Va0 and Va40), as shown in Figure 8a,f, features larger pores of about 50–70 μm randomly dispersed within the sample, leading to an increased porosity of 37–42%. The weak planes occur as a network between the pores, explaining the easy failure of the samples under compressive effort. The second statistical group (Va30 and Va35) has a better organization of the filler particles, with the kaolinite matrix generating pores predominantly of 20–30 μm, ensuring a total porosity of 21 to 24%, as shown in Figure 8d,e. The weak planes are shorter and well dispersed within the kaolinite matrix. This ensures better resistance under compression, confirming the observations made in the SEM images in Figure 7d,e. The third statistical group (Va20 and Va25) features the best compactions achieved with vibration, resulting in significant reduction in the pore size. The dominant fraction ranges from about 10 μm with a lower total porosity of 10–15%, as shown in Figure 8b,c. The pore disposal within the fracture surface relates to kaolinite matrix excoriation during the breaking process. These are most likely formed during the relative interpenetration of the filler particles (quartz and pre-existent mullite) with small kaolinite platelets with sizes of 2–5 μm according to the data in Table 2. Small differences occur between samples Va20 and Va25, which have irregular patterns of the pore’s distribution intercalated with small weak planes, as shown in Figure 8b. On the other hand, Figure 8c reveals a uniform distribution of the finest pores in the Va25 sample and the complete lack of weak planes.

**Figure 9:**
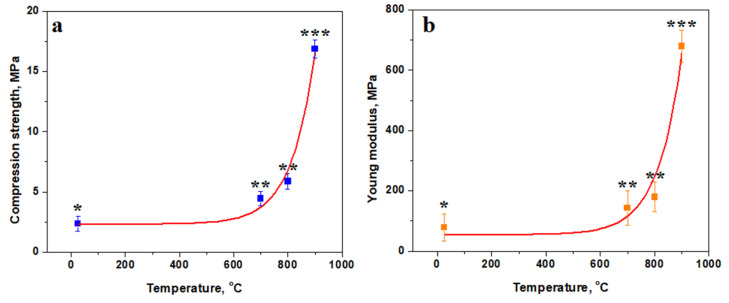
Variation in mechanical properties with the firing temperature: (**a**) compression strength and (**b**) Young’s modulus. The relevant statistical groups are marked with (*) first group, (**)second group and (***) third group. The red lines are the variation trend.

The results show that Va25 has the best mechanical characteristics as a green sample, and it should be further utilized for thermal treatments. The variation in mechanical properties with treatment temperature is presented in Figure 9. Compressive strength exhibits reversed asymptotic variation with a slow increase from 25 to 700 °C, demonstrating an inflexion region between 700 and 800 °C and a faster increase up to 900 °C (Figure 9a).

The slow increasing region is characterized by the consolidation of kaolinite matrix induced by the starting of the dehydration process, which increases the cohesion with the filler particles. The inflexion region is influenced by the complete dehydroxylation of the kaolinite matrix, turning it into a dense structure. It ensures better cohesion of the filler particles, which resists better under compressive strength. The faster increase in the compression strength is promoted by the advanced diffusion occurring between the pre-existent mullite particles and the dehydroxylated kaolinite matrix by forming new mullite bridges interconnecting the filler particles in a tight network. The statistical analysis reveals significant differences in the mean value of compressive strength at each tested temperature, proving that it is a dynamic process.

The statistical analysis reveals that Va25 sample unexposed to the heat treatment constitutes the first statistical representative group in Figure 9, representing the low values for the compressive strength and Young’s modulus. The samples heated at 700 and 800 °C form the second relevant statistical group (*p* > 0.05), revealing a significant increase in compressive strength and elastic properties as consequence of dehydration of the kaolinite matrix. The partial mullitization of the dehydrated kaolinite mixture increases the Va25/900 sample cohesion and its mechanical properties, forming the third statistical relevant group represented by a higher compression strength and elastic modulus. ANOVA test followed by Tukey post hoc tests reveal significant statistical differences among these three groups (*p* < 0.05).

The thermal consolidation of the samples is effective for improving the elastic behavior of the material, as shown in Figure 9b. The variation curve also has a reversed asymptotic trend with a small increase at lower temperatures and high increase in the Young’s modulus at higher temperatures. Sample elasticity is ensured by the strong cohesion of the matrix embedded with filler particles. The mullite diffusion necks developed in Va25/900 ensure a very robust microstructure that has increased elastic behavior, making a tenacious material.

**Figure 10:**
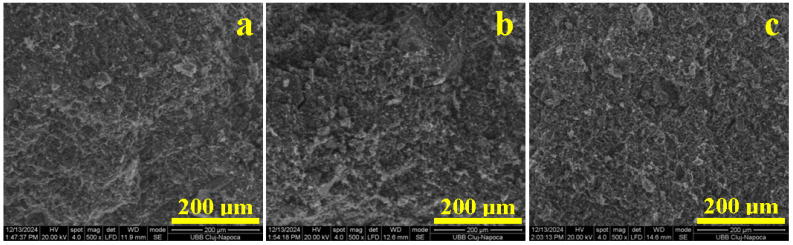
SEM images of the fracture surface of the heat treated samples: (**a**) Va25/700, (**b**) Va25/800, and (**c**) Va25/900.

The fracture surface of the Va25/700 sample is smooth and uniform, revealing the failure of matrix layers under compressive effort that cleaves the incomplete dehydrated kaolinite foils, as shown in Figure 10a. In fact, the failure mechanism remains similar to Va25 at this stage.

The partial densification of the matrix ensures the relative slow increase in the compressive strength. Complete dehydroxylation of the kaolinite matrix within Va25/800 achieves a strong consolidation of the filler particles that support higher compressive efforts, as shown in Figure 10b. The axial solicitation is dispersed through the filler particles and dissipated through the toughened dehydroxylated kaolinite, resisting lateral swelling. It has a rather fragile break at the peak load, and the cracks are promoted vertically through the sample, causing its failure. The brittle failure is sustained by dimples that are 20–50 μm in diameter, suggesting the places where the filler particles were suddenly pulled out by the dissipated effort.

The fracture surface microstructure has a drastic change in the Va25/900 sample because of the dense embedding of the filler particles into a complex matrix. The effort dissipated through the filler particles is discharged through the mullite necks, which resist the progressive load. The tight surrounding dehydroxylated kaolinite matrix ensures the background support for the mullite necks, which finally break at the peak load, creating large, rounded dimples of about 75 μm in diameter such as the one observed in the middle of Figure 10c. The neck failure directly exposes the dehydroxylated kaolinite matrix to the effort causing its brittle failure with a smooth surface. It is a characteristic of a tenacious material that is ideal for use in construction.

**Figure 11:**
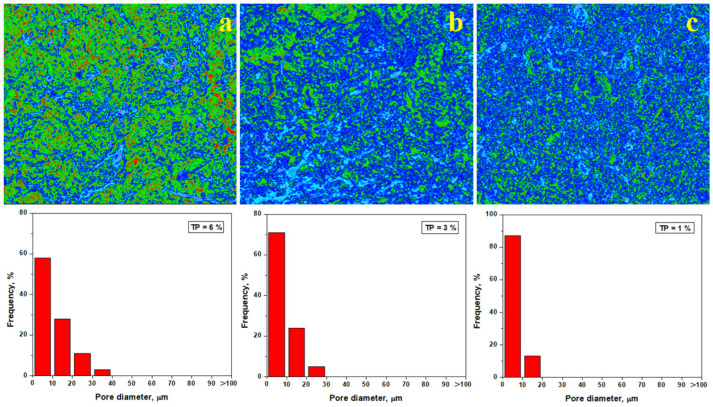
Pore distribution within the fracture surface of the heat treated samples: (**a**) Va25/700, (**b**) Va25/800, and (**c**) Va25/900.

Pore evolution plays an important key role during thermal consolidation. Pore quantification of the heat-treated samples using Image J software is presented in Figure 11. A significant decrease in the porosity to about 6% is observed after heat treatment at 700 °C, and pore diameter (about of 57% of pores being situated below 10 μm) also decreases as observed in Figure 10a. The kaolinite matrix (green nuance) surrounds the filler particles (blue). However, the dehydration process is only initiated, and major structural changes were not revealed. This finding is in good agreement with mineralogical optical microscopy images shown in Figure 3b.

The total porosity decreases down to about 3% after firing at 800 °C, as shown in Figure 11b, and the pore diameter decreases with the smaller fraction being situated around 70%. The complete dehydration of the kaolinite matrix changes the sample’s consistency. This is shown in blue, indicating its strong consolidation on the filler particles. This major change influences the failure mode, causing a change from the plastic mode to brittle failure. This is indicated by the advanced transformation of the green areas into blue, as shown in Figure 11b. The partial mullitization of the dehydrated kaolinite matrix revealed using XRD and mineralogical optical microscopy is also proven by the pore’s evolution in Figure 11c. Their size strongly decreases; about 83% of them are situated below 10 μm, ensuring a total porosity of about 1%. This represents a strong consolidation of the matrix onto the filler particles. The partial mullitization of the dehydrated kaolinite matrix enhances the sintering necks developed between microstructural constituents, making the sample stronger. Thus, the failure mode becomes brittle.

## 4. Discussion

All industrial facilities fabricating ceramic tiles have the same problem regarding wastewater contamination with mineral particulate matter dispersion and the resulting slurry after the pressing filtering [41,42]. Their dumping in landfills presents the potential hazard of emitting PM2.5 and PM10 fractions (PM2.5 are particles dispersed in atmosphere having diameter of 2.5 μm and below; similarly, PM10 particles have a maximum diameter of 10 μm) into atmosphere if they are not properly wetted [43]. Of course, the dumping and wetting processes are costly and reduce the sustainability of the process. The presence of iron hydroxide affects its re-circulation in the same technological process because the iron-related stain affect the glaze [12,13], but the mechanical properties are not affected. However, some new approaches use a waste mixture of steel slag and broken brick to develop a new pyroxene ceramic system that embeds iron oxides in the composite structure, avoiding the risk of stains [44]. Unfortunately, such approach is not available for the classical alumino-silicate ceramics based on the kaolinite–mullite system. Therefore, economic efficiency requires finding sustainable recycling solutions, which can transform potentially hazardous waste into raw materials for a byproduct.

Our previous study discusses all primary aspects regarding the resulting slurry from the wall and floor tile production wastewater [15,16]. Consolidation of this waste with a low carbon footprint was identified as possible way for the sustainable recycling of this waste to make ecological bricks. The carbon footprint is affected by so many parameters that generate CO_2_ that is released into the atmosphere, such as energy consumption, transportation of raw materials and final products, technological CO_2_ emissions, waste management related energy consumption, and hazardous emissions [45]. The used energy also has a great impact on the carbon footprint. Generally, electrical energy is considered cleaner than burning fossil fuels, but it really depends on its primary source. The electricity produced from green sources such as hydro, eolian, and tidal sources ensures an effective reduction of the carbon footprint. If the electricity is produced by burning fossil fuel, the pollution is transferred from the ceramic tile production site to the electrical energy production site, maintaining the increased carbon footprint.

As a consequence, our working hypothesis relies on controlling the technological parameters that would reduce the carbon footprint of the entire presented scenario. These are the firing temperature and the thermal treatment time. The literature data show that bricks are commonly fired at about 1000 °C to ensure clay mullitization and the subsequent desired compression strength. The literature shows that the temperature might vary from 700 to 1100 °C depending on the feedstock [18]. Thus, we choose to test our slurry behavior at 700, 800, and 900 °C, which are below the mullitization temperature. Small samples were chosen (10 mm diameter and 11 mm height) to comply with a short thermal treatment, and the sample is maintained for only 30 min at the desired temperature.

The collected slurry is quite heterogeneous because of the dumping conditions and requires proper preparations. It contains bigger quartz and mullite particles in the range of 2–100 μm mixed up with large amounts of fine kaolinite particles in the range of 1–20 μm. The moisture was adjusted at 30% using technological water (pH = 10) and milled for 10 min in a blade mill at 6000 rpm. It ensures a proper dispersion of the quartz and mullite particles regarding the finest fractions of kaolinite. Hence, the compaction load cannot be applied to ensure proper binding of the particles; the molded samples were exposed to vibration at several amplitudes. The worst results were obtained at an amplitude of 40 mm, and the best results occur at an amplitude of 25 mm. In this case, quartz and mullite particles are well dispersed and properly embedded into a dense kaolinite matrix, which ensures a compressive strength of about 2.37 MPa and a failure mode through the kaolinite binding layer that cleaves under the dissipated compression effort, promoting the sample’s cracks.

The optimal conditioned sample Va25 was further subjected to the heat treatment investigation. Dried samples were introduced into the furnace chamber at 25 °C, heated in the furnace at a rate of 10 °C/minute, and maintained for 30 min at the desired temperature. This was followed by a cooling rate of 10 °C/minute, and the sample was extracted at room temperature according to the heating diagram in Figure 1d.

The thermal consolidation mechanism is first promoted by the glass particles melting around 500–600 °C. This enhanced the matrix adhesion on the quart and pre-existent mullite particles and further promotes the kaolinite dehydroxylation process. The literature data reveal that kaolinite heating below the mullitization temperature removes O-H bonds within its structure, causing a densifying compaction [29,30]. A similar transformation is reported for metakaolin, which becomes rather amorphous by destruction of the long range order [46,47]. Our dehydroxylated kaolinite keeps the crystalline state, as evidenced by the intense XRD peaks for the dehydroxylated crystallographic planes. This explains the advanced consolidation of the samples.

A thermal treatment time of 30 min was not enough for the complete dehydroxylation of the kaolinite matrix at 700 °C, leading to a slow increase in the compressive strength and a failure of the binding layer, as shown in Figure 9a. However, 30 min of thermal consolidation was sufficient to complete the kaolinite matrix dehydroxylation at 800 °C, achieving a significant increase in the compressive strength and a typical ceramic fracture surface, as shown in Figure 9b. The obtained results reveal that pre-existent mullite filler particles have a strong interaction with the dehydroxylated kaolinite matrix, inducing local mullitization of the adhesion points that further promote diffusion necks and increasing the sample consolidation. This fact is sustained by the significant increases in the compressive strength and elasticity modulus. The advanced pores were monitored using Image J software. This analysis revealed pronounced decreases in diameter along with a significant reduction in the total porosity, indicating the strong consolidation of the filler particles within the matrix.

On the other hand, summarizing all of the evidence shows that the thermal treatment at 700 °C ensures a compressive strength comparable to the low-quality bricks. The compressive strength obtained after heating at 800 °C is comparable with regular bricks, and that obtained after treatment at 900 °C results in compressive strength comparable with the high-quality bricks. This fact has great importance for the further development of the byproduct technological process, allowing the production of ecological bricks with desired qualities. The current results were obtained using 10 mm diameter samples. This has an important implication on the brick’s design, where it is recommended that the thickness of the cellular structure of the walls should not exceed 10 mm.

It is difficult establishing an exact carbon footprint of the investigated samples. However, considering the furnace electrical energy consumption, we can estimate the heat treatment carbon footprint. Thus, the treatment effectuated at 700 °C has a lower carbon footprint than the treatment at 900 °C. In fact, it depends on the electricity source, which has a great influence on the carbon footprint. If the consumed electrical energy is green (produced by hydroelectric, photovoltaic, or eolian sources), the carbon footprint will be theoretically zero. However, if electrical energy is produced by fossil fuels, such as methane gas, it will have a significant carbon footprint. We have measured the electrical energy consumed for the thermal treatment of the samples (700, 800, and 900 °C) and extrapolate the consumption at 1100 °C. Therefore, the equivalent methane consumption can be estimated using the higher heating value of methane of 10.471 kWh/m^3^, see Table 4, and taking into consideration the following methane burning equation:

CH_4_ + 2O_2_ → 2H_2_O + CO_2_↑ (1)

Thus, the amount of emitted CO_2_ can be calculated.

The resulting ceramic slurry samples with a diameter of 10 mm and height of 11 mm and treated at the normal mullitization temperature of 1100 °C have a carbon footprint of 1.5 kg of emitted CO_2_. The reduced energy consumption by sample treatment below the mullitization temperature of 1100 °C [20,21] has the benefit of significantly reducing the emitted CO_2_.

A specific carbon footprint can be calculated for our consolidated materials by reporting the sample’s carbon footprint to its weight. Thus, the Va25/700 sample has a specific carbon footprint of 0.63 kgCO_2_/g, Va25/800 has a specific carbon footprint of 0.75 kgCO_2_/g, and Va25/900 has 0.87 kgCO_2_/g. These are very useful indicators for the further design of ecological bricks, indicating the efficiency of the moderate thermal consolidation. Improving the thermal consolidation footprint will significantly reduce the overall carbon emission associated with the development of bricks produced with wastewater ceramic slurry, making them ecologically sound.

The absolute carbon footprint of the thermal consolidated samples depends on the energy used for all preparation samples, including slurry milling during the conditioning and vibration processes. These aspects are subsidiary to the main effects of the thermal consolidation and were not assessed in this research, representing a study limitation. Further research on the brick design would consider all energy demands involved in each technological step.

The reduction of ceramic slurry in landfills through its consumption as a raw material for the manufacturing of ecological bricks represents another important goal targeted by the present study. These landfills require proper moistening to keep their fine particles trapped on the dump surface. The finest pollutants, including PM1, PM2.5, and PM10, might be released into the atmosphere if the dump becomes dry [14,43]. Such emissions have very hazardous potential, causing various pulmonary affections, like silicosis [48], asthma [49], and cancer [50]. Their atmospheric re-suspension affects the landfill vicinity and is collected by the vehicles passing through the affected area, which further spreads it to other places [51]. The mitigation of such hazards is costly and mandatory and increases the landfill owner’s expenses. The utilization of the wastewater ceramic slurry as a raw material reduces the owner’s environmental expenses and produces profit through its industrial valorization.

## 5. Conclusions

Reducing the firing temperature and treatment time reduces the sample’s carbon foot. Glass particle melting activates the thermal consolidation mechanism, enhancing the binding of the kaolinite matrix to the quartz and pre-existing mullite particles, and continues further through kaolinite dehydroxylation. The strong embedding of pre-existent mullite particles into the dehydrated kaolinite matrix facilitates the partial mullitization of the adhesion points at 900 °C, promoting diffusion necks that enhance the sample’s mechanical properties. Samples fired at 700 °C ensure a compressive strength of 4.44 MPa comparable to low-quality bricks but yield a strong reduction in the thermal consolidation carbon footprint to 630 tonCO_2_/ton. Samples fired at 800 °C ensure a strength of 5.88 MPa comparable to regular bricks with a carbon footprint of 750 tonCO_2_/ton. Samples fired at 900 °C ensure a superior strength of 16.87 MPa comparable to high-quality bricks with a specific carbon footprint of the thermal consolidation of 870 tonCO_2_/ton. Their carbon footprint is significantly below the bricks fired at the mullitization temperature.

## Figures and Tables

**Table 1:** Sample preparation characteristics.

Sample Code	Vibration		Drying Time, Months	Firing Parameters			
	Amplitude, mm	Time, min		Heating Rate, °C/min	Temperature, °C	Time, min	Cooling Rate, °C/min
Va0	0	10	3	-	-	-	-
Va20	20	10	3	-	-	-	-
Va25	25	10	3	-	-	-	-
Va30	30	10	3	-	-	-	-
Va35	35	10	3	-	-	-	-
Va40	40	10	3	-	-	-	-
Va25/700	25	10	3	10	700	30	10
Va25/800	25	10	3	10	800	30	10
Va25/900	25	10	3	10	900	30	10

**Table 2:** Mineral composition of ceramic slurry samples.

Sample		Mineral Composition				
		Quartz	Kaolinite	Mullite	Lepidocrocite	Hematite
Va25	Amount, %	36	38	17	9	-
	Size, μm	2–80	1–15	5–100	2.5–25	-
Va25/700	Amount, %	37	36	19	-	9
	Size, μm	2–80	2–15	10–150		3–30
Va25/800	Amount, %	37	33	21	-	9
	Size, μm	2–100	2–20	20–180		5–30
Va25/900	Amount, %	36	31	25	-	8
	Size, μm	2–80	5–20	25–220	-	5–30

**Table 3:** Elemental composition of the samples’ microstructure.

Sample	Elements, at.%							
	O	Si	Al	K	Fe	Ca	Na	Mg
Va25	72.7	15.2	6.6	0.8	0.3	2.0	1.9	0.5
Va25/700	69.0	17.4	7.1	1.1	0.6	2.4	2.1	0.3
Va25/800	72.3	16.1	6.8	0.7	0.3	1.3	2.2	0.3
Va25/900	69.6	17.8	7.1	0.8	0.4	1.7	2.2	0.4

**Table 4:** The carbon footprint of the thermal consolidation process.

Temperature, °C	700	800	900	1100
Energy consumption, kW/h	15.00	17.14	19.28	23.57
Equivalent gas consumption, m^3^	1.43	1.63	1.84	2.25
CO_2_ amount, kg	0.95	1.09	1.23	1.50
Specific carbon footprint, kgCO_2_/g	0.63	0.75	0.87	-
Specific carbon footprint, tonCO_2_/ton	630	750	870	-

Note: The specific carbon footprint is given per gram or per ton of heated slurry.

## Data Availability

The original contributions presented in this study are included in the article. Further inquiries can be directed to the corresponding author.

## Abbreviations

The following abbreviations are used in this manuscript:

AFM	Atomic Force Microscopy
EDS	Energy Dispersive Spectroscopy
MOM	Mineralogical Optical Microscopy
SEM	Scanning Electron Microscopy
XRD	X-ray Diffraction

## References

[1] Liu G., Li Y., Hou J., Wang Y., Lin D. (2025). A review on the industrial waste based adsorbents for the removal of pollutants from water: Modification methods and adsorption study, Resources. Environ. Sustain..

[2] Normazlan W.M.D.W., Buthiyappan A., Jais F.M., Raman A.A.A. (2025). Exploring the potential of industrial and municipal wastes for the development of alternative fuel source: A review. Process Saf. Environ. Prot..

[3] Peng L., Fan Z., Zhang X. (2025). Consumer Orientation and Market-Driven Strategies for Promoting Low-Carbon Innovation in Supply Chains: Pathways to Sustainable Development. Sustainability.

[4] Kim S., Cha H., Lee T., Kim J.Y., Lee J., Jang S.-H., Kwon E.E. (2025). Suppression of carbon footprint through the CO2-assisted pyrolysis of livestock waste. Sci. Total Environ..

[5] Paleologos E.K., Mohamed A.-M.O., Mohamed D., Al Nahyan M.T., Farouk S., Singh D.N. (2025). Decarbonization of the Waste Industry in the U.S.A. and the European Union. Sustainability.

[6] Casapino-Espinoza C.A., Gómez-Soberón J.M., Gómez-Soberón M.C. (2025). The Effect of Recycled Crushed Brick Aggregate on the Physical–Mechanical Properties of Earth Blocks. Buildings.

[7] Chen Z., Kurniawan T.A., Yap P.-S. (2024). Integrating leachate treatment into circular economy landfill practices for nutrient, energy, and material (NEM) recovery and climate change mitigation. J. Water Process Eng..

[8] Ishkov A.G., Zhdaneev O.V., Romanov K.V., Koloshkin E.A., Kulikov D.V., Mikhailov A.M., Dzhus K.A., Lugvishchuk D.S., Bogdan I.B., Maslova E.V. (2024). Methodological approaches to carbon footprint assessment and certification of low carbon hydrogen. Int. J. Hydrogen Energy.

[9] Al Mubarak F., Rezaee R., Wood D.A. (2024). Economic, Societal, and Environmental Impacts of Available Energy Sources: A Review. Eng.

[10] Ciacco E.F.S., Rocha J.R., Coutinho A.R. (2017). The energy consumption in the ceramic tile industry in Brazil. Appl. Therm. Eng..

[11] Lu L., Chen Y., Feng Q., Li W., Chen D. (2024). Long-range energy demand and greenhouse gas emissions analysis using the LEAP Model: A case study of building ceramic industrial park. Energy Sustain. Dev..

[12] Mourou C., Zamorano M., Ruiz D.P., Martín-Morales M. (2023). Characterization of ceramic tiles coated with recycled waste glass particles to be used for cool roof applications. Constr. Build. Mater..

[13] Wang Y., Yu S., Chu J., Chen D., Chen J. (2018). Study on the copper and iron coexisted coloring glaze and the mechanism of the fambe. J. Eur. Ceram. Soc..

[14] Mizutani R.F., de Paula Santos U., Arbex R.F., Arbex M.A., Terra-Filho M. (2023). An Evaluation of the Impact of Air Pollution on the Lung Functions of High School Students Living in a Ceramic Industrial Park Zone. Int. J. Environ. Res. Public Health.

[15] Avram S.E., Barbu Tudoran L., Cuc S., Borodi G., Birle B.V., Petean I. (2024). Microstructural Investigations Regarding Sustainable Recycling of Ceramic Slurry Collected from Industrial Waste Waters. Sustainability.

[16] Avram S.E., Birle B.V., Tudoran L.B., Borodi G., Petean I. (2024). Investigation of Used Water Sediments from Ceramic Tile Fabrication. Water.

[17] Avram S.E., Tudoran L.B., Cuc S., Borodi G., Birle B.V., Petean I. (2024). Natural Fiber Reinforcement of Ceramic Slurry Compacts. J. Compos. Sci..

[18] Karaman S., Ersahin S., Gunal H. (2006). Firing temperature and firing time influence on mechanical and physical properties of clay bricks. J. Sci. Ind. Res..

[19] Dubois J., Murat M., Amroune A., Carbonneau X., Gardon R. (1995). High-temperature transformation in kaolinite: The role of the crystallinity and of the firing atmosphere. Appl. Clay Sci..

[20] Badanoiu A.-I., Stoleriu S.-P., Carocea A.-C., Eftimie M.-A., Trusca R. (2025). Influence of Synthesis Route on Composition and Main Properties of Mullite Ceramics Based on Waste. Materials.

[21] Valášková M., Blahůšková V., Edelmannová M.F., Matějová L., Soukup K., Plevová E. (2023). Clay/Fly Ash Bricks Evaluated in Terms of Kaolin and Vermiculite Precursors of Mullite and Forsterite, and Photocatalytic Decomposition of the Methanol–Water Mixture. Minerals.

[22] Cultrone G., Rodriguez-Navarro C., Sebastian E., Cazzala O., De La Torre M.J. (2001). Carbonate and silicate phase reactions during ceramic firing. Eur. J. Mineral..

[23] Bennadji F.G., Beneu B., Laval J.P., Blanchart P. (2008). Structural transformations of Muscovite at high temperature by X-ray and neutron diffraction. Appl. Clay Sci..

[24] Rehman M.U., Ahmad M., Rashid K. (2020). Influence of fluxing oxides from waste on the production and physico-mechanical properties of fired clay brick: A review. J. Build. Eng..

[25] Santana I.S.A., Novaes M.d.P., Araújo R.C.C.d., Batalha-Vieira L. (2024). Exposed Clay Bricks Made with Waste: An Analysis of Research and Technological Trends. Sustainability.

[26] Zhang L. (2013). Production of bricks from waste materials—A review. Constr. Build. Mater..

[27] Medeiros-Junior R.A., Thiesen M., Betioli A.M., Casali J.M., Trentin L.F.Z., Frare A., Borçato A.G. (2024). Influence of Precursor Particle Size and Calcium Hydroxide Content on the Development of Clay Brick Waste-Based Geopolymers. Minerals.

[28] Catalin S., Daniela M.L., Moldovan M., Monica P.L., Borodi G., Petean I., Sorin L. (2024). Recycled Aggregates Influence on the Mechanical Properties of Cement Lime-Based Mortars. Materials.

[29] Kenzhaliyev B., Biryukova A., Dzhienalyev T., Panichkin A., Imbarova A., Uskenbaeva A., Yusoff A.H. (2024). Assessment of Microsilica as a Raw Material for Obtaining Mullite–Silica Refractories. Processes.

[30] Rocha D.R., Barber X., Jordán-Vidal M.M., Urbano A., Melquiades F.L., Thomaz E.L., Mataix-Solera J. (2022). Multivariate Analysis with XRD Data as a Fingerprinting Technique to Study Burned Soils. Minerals.

[31] Tang B., Wang G., Zhuo L., Ge J., Cui L. (2006). Facile Route to α-FeOOH and α-Fe_2_O_3_ Nanorods and Magnetic Property of α-Fe_2_O_3_ Nanorods. Inorg. Chem..

[32] Marinho J.Z., Montes R.H.O., Moura A.P., Longo E., Varela J.A., Munoz R.A.A., Lima R.C. (2014). Rapid preparation of α-FeOOH and α-Fe_2_O_3_ nanostructures by microwave heating and their application in electrochemical sensors. Mater. Res. Bull..

[33] Zurauskiene R., Navickiene L. (2023). Foam Glass Granule Usage in Tile Glue Mixtures That Use a Reduced Portland Cement Amount. Materials.

[34] Ibrahim M.H., Mustaffar M.I., Ismail A.M., Ismail S.A., Othman N. (2023). Development of porous glass-ceramic using silica sand for wall tiles application. Mater. Today Proc..

[35] Hamza A., Hussein I.A., Mahmoud M., Hussein I.A., Mahmoud M. (2023). Chapter 1—Introduction to reservoir fluids and rock properties. Developments in Petroleum Science.

[36] Zhang M., Fan X., Zhang Q., Yang B., Zhao P., Yao B., He L. (2021). Influence of multi-planes of weakness on unstable zones near wellbore wall in a fractured formation. J. Nat. Gas Sci. Eng..

[37] Wu W., Wang T., Bai J., Liu J., Wang X., Xu H., Feng G. (2024). Failure Characteristics and Cooperative Control Strategies for Gob-Side Entry Driving near an Advancing Working Face: A Case Study. Processes.

[38] Godyń K., Dutka B., Tram M. (2023). Application of Petrographic and Stereological Analyses to Describe the Pore Space of Rocks as a Standard for the Characterization of Pores in Slags and Ashes Generated after the Combustion of Municipal Waste. Materials.

[39] Hu Z., Zhang R., Zhu K., Li D., Jin Y., Guo W., Liu X., Zhang X., Zhang Q. (2023). Probing the Pore Structure of the Berea Sandstone by Using X-ray Micro-CT in Combination with ImageJ Software. Minerals.

[40] Dong Z., Tian S., Xue H., Lu S., Liu B., Erastova V., Chen G., Zhang Y. (2025). A novel method for automatic quantification of different pore types in shale based on SEM-EDS calibration. Mar. Pet. Geol..

[41] Raffaeli R., Pazzi L., Pellicciari M. (2024). Industry 4.0 Solutions as Enablers for the Sustainability of the Italian Ceramic Tiles Sector. Sustainability.

[42] Zheng T., Ardolino M., Bacchetti A., Perona M. (2023). The road towards industry 4.0: A comparative study of the state-of-the-art in the Italian manufacturing industry. Benchmarking Int. J..

[43] Pallares S., Jordan M.M., Soriano A., Vicente A.B., Pardo F., Sanfeliu T. (2011). Monitoring of As, Cd and Ni in PM10 and topsoils in a ceramic cluster. J. Geochem. Explor..

[44] Ji Y., Li E., Zhu G., Wang R., Sha Q. (2024). Preparation and Performance of Ceramic Tiles with Steel Slag and Waste Clay Bricks. Materials.

[45] Li Z., Tang P., Wang X., Liu X., Mou P. (2025). PCF-RWKV: Large Language Model for Product Carbon Footprint Estimation. Sustainability.

[46] White C.E., Kearley G.J., Provis J.L., Riley D.P. (2013). Inelastic neutron scattering analysis of the thermal decomposition of kaolinite to metakaolin. Chem. Phys..

[47] Wan Q., Rao F., Song S. (2017). Reexamining calcination of kaolinite for the synthesis of metakaolin geopolymers—Roles of dehydroxylation and recrystallization. J. Non-Cryst. Solids.

[48] Seaton A., Legge J.S., Henderson J., Kerr K.M. (1991). Accelerated silicosis in Scottish stonemasons. Lancet.

[49] Zhou X., Sampath V., Nadeau K.C. (2024). Effect of air pollution on asthma. Ann. Allergy Asthma Immunol..

[50] Xu L., Ma W., Huo X., Luo J., Li R., Zhu X., Kong X., Zhao K., Jin Y., Zhang M. (2024). New insights into the function and mechanisms of pi RNA PMLCPIR in promoting PM2.5-induced lung cancer. J. Adv. Res..

[51] Avram S.E., Tudoran L.B., Borodi G., Filip M.R., Ciotlaus I., Petean I. (2025). Physicochemical Aspects Regarding the Sustainable Conversion of Carwash Slurry as Coverage Admixture for Landfills. Sustainability.

